# Spherical alveolar shapes in live mouse lungs

**DOI:** 10.1038/s41598-023-32254-8

**Published:** 2023-03-31

**Authors:** Min Woo Kim, Byung Mook Weon, Jung Ho Je

**Affiliations:** 1 School of Interdisciplinary Bioscience and Bioengineering, Pohang University of Science and Technology, San 31, Hyojadong, Pohang, 37673 South Korea; 2 Soft Matter Physics Laboratory, School of Advanced Materials Science and Engineering, SKKU Advanced Institute of Nanotechnology (SAINT), Sungkyunkwan University, Suwon, 16419 South Korea; 3 Research Center for Advanced Materials Technology, Core Research Institute, Suwon, 16419 South Korea; 4 Department of Materials Science and Engineering, Pohang University of Science and Technology, San 31, Hyoja-Dong, Pohang, 37673 South Korea; 5 Nanoblesse Research Lab., Nanoblesse, 4Th Fl. 85-11, Namwon-Ro, Pohang, 37883 South Korea

**Keywords:** X-ray tomography, Respiration, Three-dimensional imaging, Tomography

## Abstract

Understanding how the alveolar mechanics work in live lungs is essential for comprehending how the lung behaves during breathing. Due to the lack of appropriate imaging tools, previous research has suggested that alveolar morphologies are polyhedral rather than spherical based on a 2D examination of alveoli in fixed lungs. Here, we directly observe high-resolution 3D alveoli in live mice lungs utilizing synchrotron x-ray microtomography to show spherical alveolar morphologies from the live lungs. Our measurements from x-ray microtomography show high sphericity, low packing density, big alveolar size, and low osmotic pressure, indicating that spherical alveolar morphologies are natural in living lungs. The alveolar packing fraction is quite low in live lungs, where the spherical alveoli would behave like free bubbles, while the confinement of alveolar clusters in fixed lungs would lead to significant morphological deformations of the alveoli appearing polyhedral. Direct observations of the spherical alveolar shapes will help understand and treat lung disease and ventilation.

## Introduction

Pulmonary alveoli are the most specialized and important structures in the lung for respiration and gas exchange. Direct visualization of high-resolution 3D alveolar shapes in live lungs is essential to understand the alveolar behaviors during respiration, which is required for applying proper mechanical ventilation to patients^1^ and providing fundamental information on pulmonary diseases^2^. Specifically, most critical lung diseases, including coronavirus disease 2019 (COVID-19), acute respiratory distress syndrome (ARDS), and pulmonary fibrosis, could destroy alveolar shapes so that the lungs cannot function as normal^3,4^.

For a long time (> 40 years), alveolar shapes have been primarily known to be polyhedral based mainly on the morphology of fixed lungs^5,6^. In the histological images, alveolar shapes are polyhedral in form with flat walls^5,7^. The polyhedral shape has been explained in terms of the balance of forces at an alveolar wall intersection, assuming that all alveoli are ventilated as uniformly as possible^7^. However, chemical processes for the fixation of lungs have been known to induce morphological distortion to bio-specimen, such as tissue shrinkage by dehydration^8–10^. For this reason, it is not yet clear whether the alveolar shape is polyhedral or not in live lungs^11^. The most challenging problem for visualizing high-resolution 3D alveoli in live lungs is lung movement by respiration during imaging, which induces critical noises in the images. This study aims to reveal alveolar shapes by visualizing 3D alveoli in a live lung. To overcome the technical challenge of lung movement, the tracking x-ray microscopy (TrXM) we developed in the previous study^12^ is appropriate for visualizing 3D alveoli in live intact lungs.

In this study, we successfully obtain high-resolution 3D alveolar images from live and fixed mouse lungs and confirm that alveoli seem spherical in the live lungs at the end of expiration and polyhedral in the fully inflated fixed lungs. We also estimate individual alveoli's osmotic and Laplace pressures based on 3D rendered image data to reveal how the pressures affect the alveolar shape, a well-known principle in soft matter physics. Our results show that osmotic and Laplace pressures could determine the alveolar shape by the competition between two pressures, depending on the lung environment. This study indicates that synchrotron x-ray microtomography is a promising approach to understanding alveolar mechanics and pulmonary diseases.

## Results

### X-ray imaging of 3D alveoli

**Figure 1:**
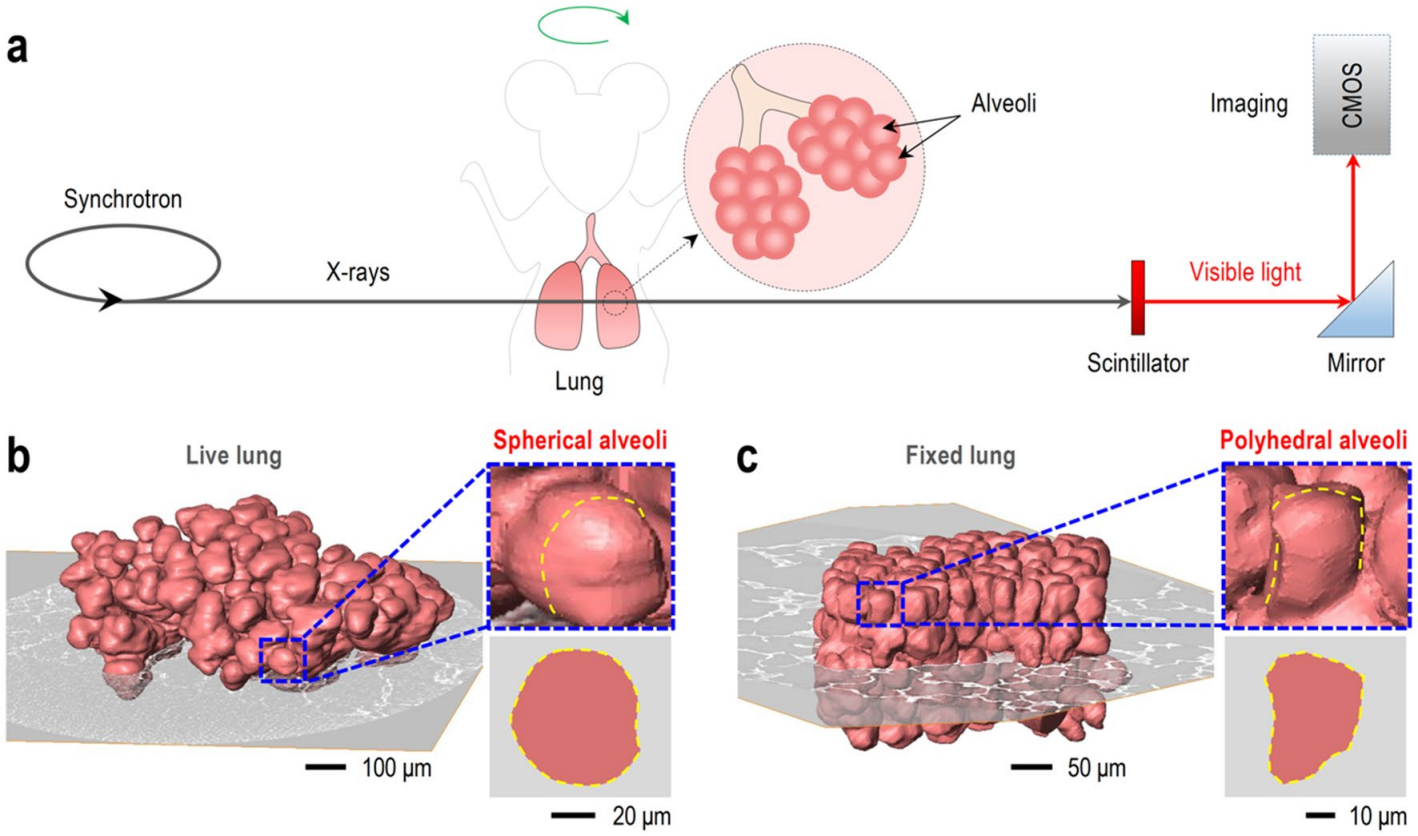
Visualization of 3D alveoli in a live and fixed mouse lung. (**a**) Schematic illustration of synchrotron x-ray microtomography for visualization of 3D alveoli in a live or fixed mouse lung. (**b**) Spherical alveoli in the live lung at expiration. (**c**) Polyhedral alveoli in the fixed lung.

To visualize 3D alveoli in live or fixed whole lungs, high spatial resolution on micrometer scales (~ 1 μm in this study) and temporal resolution on millisecond scales (5 ms/image in this study) are required with the high penetration capability to minimize the motion-blurring of alveoli. Conventional optical microscopy is limited to 2D analysis with visible light (wavelength = 380 ~ 750 nm, energy = 2.00 ~ 2.75 eV)^13^ imaging, accompanied by histological sample preparation such as chemical fixation and sectioning (penetration limits of visible lights = 0.5 ~ 2.5 mm)^14^. X-ray imaging based on synchrotron hard x-rays (> 5 keV of photon energy) obtains high-quality images for biological specimens, as required here^15–17^. We adopt synchrotron x-ray at 15 ~ 20 keV of photon energy (penetration depths > 1 cm)^18^, which provides high spatial and temporal resolution^15^ with strong penetration capability, as schematically illustrated in Fig. 1a.

The alveolar shape could be analyzed by visualizing 3D alveoli with synchrotron x-ray tomography. We successfully reconstruct 3D alveoli from the projection images taken during rotation of the specimen (green arrow in Fig. 1a, see methods for detail) and visualize the exact and precise alveolar shape in a live and the fixed lung (Fig. 1b and c).

### Sphericity of 3D alveoli

Visualization of high-resolution 3D alveoli enables us to directly compare geometric information of the alveoli in the live and fixed lungs for the first time. The alveolar shape in a live lung at the end of expiration is shown as spherical compared with polyhedral alveoli in the fully inflated fixed lung, as demonstrated in Fig. 1b and c, even though both lungs are obtained from the same mouse. Furthermore, the spherical alveolar shapes in other live mouse lungs taken by 3D tomographic images are consistent with the previous observations from 2D projection images^19^, as shown in Supplementary Fig. S1.

Quantitatively, the sphericity of the alveoli is measured from the volume (*V*_*a*_) and the surface area (*S*_*a*_) of individual alveoli:

1 $$ {\text{Sphericity}} \left( \upvarphi \right) = \frac{{\pi^{\frac{1}{3}} \left( {6V_{a} } \right)^{\frac{2}{3}} }}{{S_{a} }} $$

**Figure 2:**
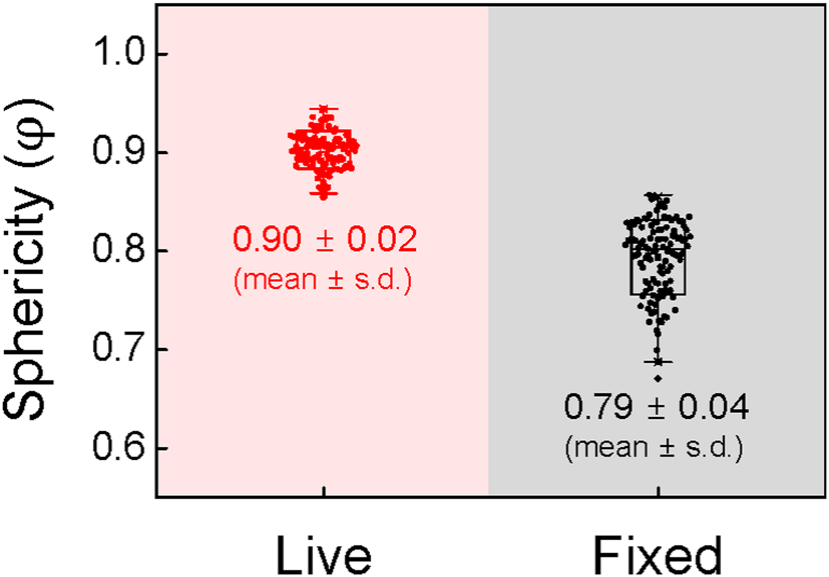
Sphericity of 120 alveoli in the live lungs and 120 alveoli in the fixed lungs. $\mathrm{\varphi }$ is the sphericity of individual alveoli ($\mathrm{\varphi }$ of the sphere = 1). $\mathrm{\varphi }$ = 0.90 ± 0.02 (mean ± s.d.) for the live lungs and $\mathrm{\varphi }$ = 0.79 ± 0.04 (mean ± s.d.) for the fixed lungs were taken from x-ray microtomography (*P*-value = 2.8 × 10^–67^). Every single measurement is not from the same alveolus.

The sphericity of 120 alveoli in 3 live lungs (40 alveoli/lung) is measured as φ = 0.90 ± 0.02 (mean ± s.d.) and higher than that of fixed lungs as φ = 0.79 ± 0.04 (mean ± s.d.) (*P*-value = 2.8 × 10^–67^ between live and fixed lungs), as summarized in Fig. 2 and Supplementary Table S1. This measurement indicates that the alveolar morphologies in the live lungs are more spherical than in the fixed lungs, which is supported by the quantitative measurement of the volume (*V*_*a*_) and the surface area (*S*_*a*_) of the individual alveoli taken from x-ray microtomographic images of the alveoli.

### Packing density of 3D alveoli

To get insight into the higher sphericity of alveoli in the live lungs, we quantitatively compare the alveolar configuration situations in the live or fixed lungs by measuring the packing density of the alveoli. The alveolar packing fraction is achievable from the volume information in x-ray microtomography, as summarized in Table 1 (by randomly selecting specific volumes at the apex of lungs, as illustrated in Supplementary Fig. S2). X-ray microtomographic 3D visualization of individual alveoli enables us to estimate the alveolar volume fraction (ϕ) of the lungs, defined as ϕ = V_airspace_/V_cube_ (here V_airspace_ is the volume of the airspace inside the cubes and V_cube_ is the volume of the cubes in the lung), providing the volume information of gas and tissue in the lungs. In detail, the ϕ values of the five random regions with cube volumes (see the inset of Supplementary Fig. S2 from 100 × 100 × 100 voxels) are selected in each lung to statistically estimate the ϕ values of the total lungs. The estimated ϕ values of the live lung (ϕ = 0.66 ± 0.02 (mean ± s.d.)) are quite smaller than that of the fixed lung (ϕ = 0.85 ± 0.03 (mean ± s.d.)), as shown in Table 1.

**Table 1:** Alveolar volume fraction (ϕ) in the live and the fixed lungs.

Cube No. in 3 live lungs	V_cube_	V_airspace_	ϕ	Cube No. in 3 fixed lungs	V_cube_	V_airspace_	ϕ
1	11,239,424	7,334,734	0.65	1	2,299,968	1,955,032	0.85
2	11,239,424	7,918,793	0.70	2	2,299,968	2,003,078	0.87
3	11,239,424	7,227,658	0.64	3	2,299,968	2,027,650	0.88
4	11,239,424	7,234,614	0.64	4	2,299,968	1,965,984	0.85
5	11,239,424	7,731,728	0.69	5	2,299,968	1,873,721	0.81
6	11,239,424	7,469,858	0.66	6	2,299,968	2,037,984	0.89
7	11,239,424	7,553,184	0.67	7	2,299,968	1,845,140	0.80
8	11,239,424	7,858,547	0.70	8	2,299,968	1,886,994	0.82
9	11,239,424	7,556,984	0.67	9	2,299,968	1,982,583	0.86
10	11,239,424	7,256,657	0.65	10	2,299,968	1,839,865	0.80
11	11,239,424	7,197,090	0.64	11	2,299,968	1,919,030	0.83
12	11,239,424	6,786,032	0.60	12	2,299,968	1,947,159	0.85
13	11,239,424	7,365,432	0.66	13	2,299,968	2,029,154	0.88
14	11,239,424	7,494,899	0.67	14	2,299,968	1,886,979	0.82
15	11,239,424	7,251,825	0.65	15	2,299,968	1,946,242	0.85

V_cube_: volume of the cubes in the lung (μm^3^) and V_airspace_: volume of the airspace inside the cubes (μm^3^), ϕ: alveolar volume fraction of the cubes, defined as ϕ = V_airspace_/V_cube_ and measured as ϕ = 0.66 ± 0.02 (mean ± s.d.) for the live lung and ϕ = 0.85 ± 0.03 (mean ± s.d.) for the fixed lung.

We also measure the maximum diameter in 3D alveoli for the live and fixed lungs, respectively from 120 alveoli in 3 live and fixed mice lungs. Our measurements as *D* = 35.9 ± 7.2 μm (mean ± s.d.) of the fixed lungs, resembling densely packed clusters of alveoli (Fig. 1c and Supplementary Table S2), are much smaller than *D* = 81.6 ± 18.2 μm (mean ± s.d.) for those of the live lungs. The relatively smaller alveolar sizes for the fixed lungs are attributed to the fixation, leading to shrinkage from the original alveolar volumes, which would be the volumes of the live lungs. The small alveolar size corresponds to the high packing density of the fixed lungs, representing a compaction situation of randomly packed neighboring alveoli.

## Discussion

In this study, synchrotron x-ray imaging directly lets us compare alveolar morphology between live and fixed lungs. Specifically, the alveolar morphology has been reported as polyhedral^5–7^ with 40 ~ 58 μm diameter in fully inflated fixed lungs^20–22^. However, we reveal that alveolar morphology in live lungs shows spherical and 2 ~ 3 times the larger alveolar diameter (81.6 ± 18.2 μm) even at the expiration than reported in fixed lungs. These results imply that studying alveoli in live lungs are required for a more accurate understanding of lungs.

The small ϕ values (ϕ = 0.66 ± 0.02) and the large *D* values (*D* = 81.6 ± 18.2 μm) in the live lungs indicate less compaction of alveolar clusters^23^, which would be responsible for the high sphericity (φ = 0.90 ± 0.02) in the alveolar morphology in the live lungs. In contrast, the low sphericity (φ = 0.79 ± 0.04) of the alveoli in the fixed lungs corresponds to the large ϕ values (ϕ = 0.85 ± 0.03) and the small *D* values (*D* = 35.9 ± 7.2 μm). We attribute the spherical morphology of alveoli in live lungs to less compaction among alveolar clusters, which would be favorable for keeping the high sphericity that will cause less deformation of individual alveoli or less morphological interference among alveoli.

We consider the alveolar compaction situation by analogy with alveoli as bubbles. Like a bubble, the intrinsic physical force to make an alveolus spherical is the Laplace pressure between the inside and the outside of a curved interface between two fluids (air and tissue). For a spherical bubble, the pressure difference is described as *ΔP* = 2*γ*/*R* (where *γ* is the film (or tissue) surface tension and *R* is the radius of the curved surface)^23^. The confinement effect by a cluster of bubbles (or alveoli) is evaluated by the confinement pressure *P*, which corresponds to the osmotic pressure *Π* as a function of the alveolar volume fraction ϕ^23^. From observations and theoretical explanations for a cluster of bubbles^24^, we estimate *P* ~ 0.5(*γ*/*R*) at ϕ ~ 0.85 for the fixed lungs, which is comparable to *ΔP*, while *P* ~ 0.01(*γ*/*R*) at ϕ ~ 0.66 for the live lungs, which is negligible. Interestingly, we find that the Laplace pressure is more dominant than the confinement (osmotic) pressure in the live lungs (*ΔP* » *P*), which would explain the *spherical* morphology of individual alveoli in the live lungs. The confinement (osmotic) pressure in the fixed lungs, which is comparable to the Laplace pressure (*ΔP* ~ 4*P*), would induce the significant morphological deformation of individual alveoli to appear *polyhedral*, as observed previously. We note that the alveolar packing fraction is quite low in the live lungs, where the spherical alveoli would behave like free bubbles.

We successfully visualize 3D alveoli in live and fixed lungs with synchrotron x-ray microtomography to reveal whether the alveolar shape is polyhedral or spherical. Although we perform the experiments only at the expiration (Pressure $\approx$ 0 cmH_2_O) for live lungs due to time-consuming and laboring manual segmentation process (several months/segmentation for a set of tomography), our observations significantly show spherical morphology from the live lungs even at the low pressure. In the previous reports, spherical alveoli have often been suggested only under high-TLC conditions (> 80% of total lung capacity (TLC))^5,25^. We suppose that high-TLC conditions will decrease the alveolar packing density, thus diminishing the confinement effect of a cluster of alveoli. Consequently, the individual alveoli will behave like free bubbles in high-TLC conditions. In other words, we expect that the spherical alveolar shape maintains its shape at the inspiration. For further study of alveolar shape changes at the inspiration, the development of appropriate automatic segmentation or development of de-noising techniques in images^26^ are also crucial to better address the laborious work of manual segmentation.

We suggest that the different alveolar morphology in live and fixed lungs can be explained by the confinement effect of a cluster of alveoli, like a cluster of bubbles, in terms of soft matter physics. This consideration is plausible because normal lung tissues are quite soft with an extremely low Young’s elastic modulus of about 1 kPa^27^, which is enough to take tissues as soft films in a cluster of bubbles.

This study delivers quantitatively comparative evaluations of actual alveolar shapes in live and fixed lungs based on the 3D x-ray tomographic images, consistent with the previous observations in 2D histological images^5,7^ and 2D x-ray projection images^12,19^. We believe that spherical alveoli are natural in live lungs and polyhedral alveoli in fixed lungs according to the lung environment. The packing density of alveoli taken from the 3D x-ray tomographic images can be a key quantitative clue for the alveolar morphology. The confinement effect of alveolar clusters would explain the alveolar morphology. Further studies are required to generalize the confinement effect of alveoli in various lung situations.

In conclusion, we directly observe high-resolution 3D alveoli in live mice lungs at the expiration utilizing synchrotron x-ray microtomography. Direct measurements of 3D alveoli show spherical alveoli from the live lungs and polyhedral alveoli in the fixed lungs. Our measurements from x-ray microtomography show high sphericity, low packing density, big alveolar size, and low osmotic pressure, indicating that spherical alveolar morphologies are natural in living lungs, in contrast to polyhedral alveoli in fixed lungs. We suggest the confinement effect of alveolar clusters to explain the alveolar morphology. This study will help understand lung function and improve ventilation.

## Methods

### Animal preparation

All experimental protocols were approved by the SPring-8 Experimental Animals Care and Use Committee. This research followed ARRIVE guidelines and all experiments were performed in accordance with the relevant regulations. Total three eight-week-old SPF pathogen-free nude mice (BALB/c-nu, body weight: 20–25 g, male, SLC Japan Inc., Japan) were anesthetized with an injection of a mixture of medetomidine, butorphanol, and midazolam. A tracheostomy was performed by inserting a tightly secured 22 G Jelco® I.V. catheter (Johnson & Johnson Medical, USA). After surgery, the animal was mounted in the vertical position in a custom-made plastic holder and placed in the experimental hutch for imaging. The catheter was connected to a mechanical ventilator (Harvard Apparatus, USA). The lung was ventilated with the following ventilation parameters: respiratory rate = 100 breaths/min., tidal volume = 160 μl/respiration.

### Imaging based on tracking x-ray microscopy

To visualize alveolar shape in live lungs, we obtained computed tomography (CT) data of alveoli during respiration by Tracking x-ray microscopy^12^. All imaging experiments for live lungs were performed at RIKEN Coherent x-ray Optics beamline (BL29XU) at SPring-8 in Japan: http://www.spring8.or.jp for providing high spatial and temporal resolution. A mouse was mounted on a 6-axis motorized stage (Kohzu precision) using a custom-made holder. After passing through the mouse, the transmitted x-ray beam was converted through a scintillator crystal (LSO:Tb) to visible light. The images were then captured by a CMOS camera (pco.edge 5.5 CLHS, PCO AG, Germany, 2560 × 2160 pixels). Adequate pixel size was 0.56 μm in this setup and the exposure time was 5 ms.

The imaging system, such as the camera, motorized stages, and shutter, was synchronized with the mechanical ventilator to maintain lung position during exposure for imaging, as previously described in our study^12^. The synchronization between the imaging system and the mechanical ventilator was based on the TTL (transistor-transistor logic) signal. Specifically, the TTL triggered the camera exposure at every same point of the respiratory to capture only the same position (Apex of the lung at the end of expiration) during imaging. The images were obtained after 560 ms from every starting point of each respiratory cycle. The every 360 projection images were taken during a rotation of 180° at every 0.5° rotation for each tomography scan. All triggering control was done using a self-developed Visual Basic program.

### X-ray imaging for a fixed lung

After the experiment, the lungs were fixed by Heitzman’s method^28,29^ and imaged to compare alveolar shapes in live and fixed lungs. The fixative solution was made of 50% (by volume) polyethylene glycol 400, 25% ethyl alcohol (95%), 10% formaldehyde (37%), and 15% double-distilled water. The solution was gently injected through the tracheal intubation with inflating the lungs to the maximal volume. The fixed whole-lungs were dried by air blower (Pressure: under 20 cm H_2_O) for 72 h to remove all the liquid from the sample. Then, the lungs became air-filled after drying and maintained their inflated state. Imaging experiments for the fixed lung were performed at the 6C Biomedical Imaging beamline at Pohang Light Source in Korea: http://pal.postech.ac.kr/. The fixed lung was mounted on a 6-axis motorized stage (Kohzu precision) with a custom-made holder. After passing through the sample, the transmitted x-ray beam was converted by a scintillator (LSO:Tb) to visible light. The images were then captured by a CMOS camera (pco.edge 5.5 CLHS, PCO AG, Germany, 2560 × 2160 pixels). The adequate pixel size was 0.33 μm and the exposure time was 500 ms. A total of 600 projection images of the lung apex were taken during the rotation of 180° for each tomography scan.

### 3D image acquisition

After imaging, we applied the Lanczos algorithm to downsample the images to 640 × 540 pixels for manageable data size. Projection images were reconstructed using the Octopus 8.9 software (Inside Matters NV, Belgium) with a filtered back-projection algorithm. Airspaces of alveoli in reconstructed images were manually segmented and the segmented images were rendered as a 3D structure using the Amira 5.2 software (Visage Imaging, USA).

### Statistical analysis

Data were presented as mean ± s.d. (standard deviation). *P*-values were determined by performing a two-tailed t-test.

## Supplementary Information

Supplementary Information.

## Data Availability

The datasets used and/or analyzed during the current study available from the corresponding author on reasonable request.
