# Supplementary material for: Spherical alveolar shapes in live mouse lungs
Source: Sci Rep. 2023 Mar 31;13:5319. doi: 10.1038/s41598-023-32254-8 (PMC10066015; doi:10.1038/s41598-023-32254-8)
Supplement: Supplementary file 1 — Supplementary Information. [file 41598_2023_32254_MOESM1_ESM.docx]

**SUPPLEMENTARY INFORMATION**

**Spherical alveolar shapes in live mouse lungs**

Min Woo Kim, Byung Mook Weon, and Jung Ho Je

| Supplementary **Figure S1** | Projection images of alveoli in live mice lungs |
| --- | --- |
| Supplementary **Figure S2** | Measurements of alveolar gas packing fraction (φ) in lungs |
| Supplementary **Table S1** | Sphericity of 120 individual alveoli in 3 live mice lungs and 3 fixed mice lungs. |
| Supplementary **Table S2** | Diameters of 120 individual alveoli ($\mu m$) in 3 live mice lungs and 3 fixed mice lungs. |


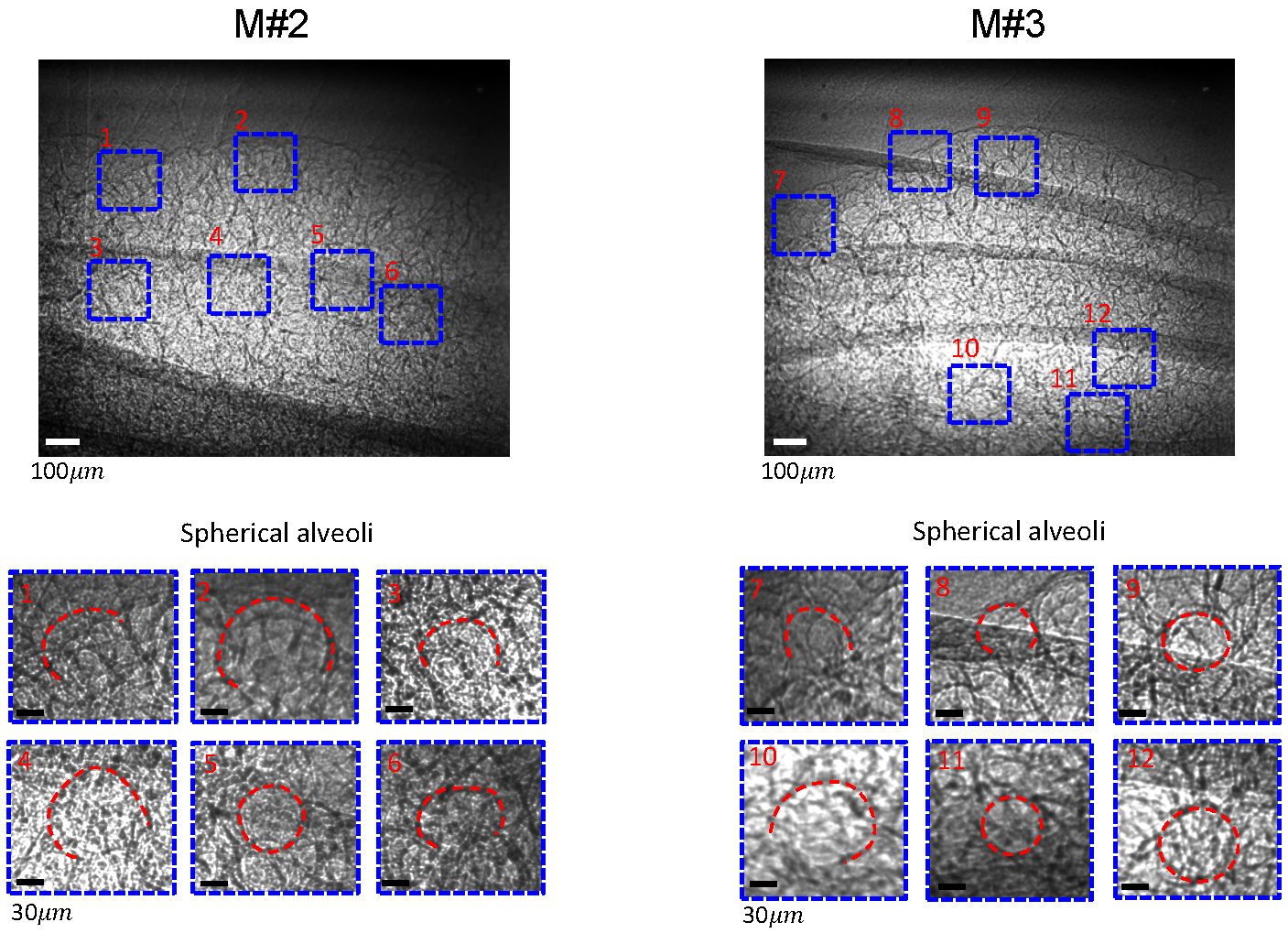


**Figure S1. Projection images of alveoli in live mice lungs.** M# means the mouse number. The 3D alveolar shape of the M#1 mouse was shown in **Figure 1** in the manuscript. Numerous alveolar boundaries of lung apex regions are shown in the upper figures. Red dashed lines indicate the spherical boundary of individual alveoli in the projection images.


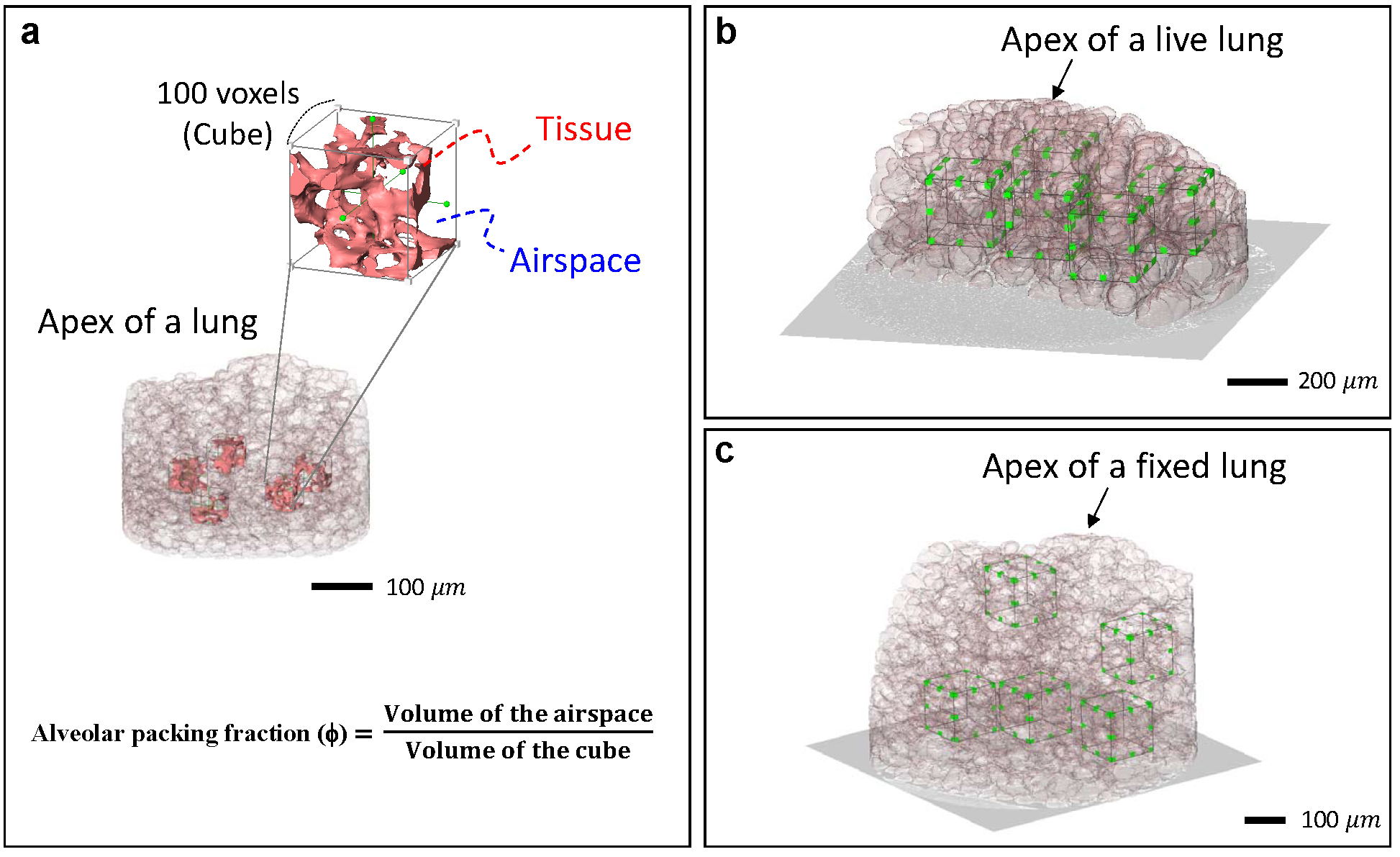


**Figure S2. Measurements of alveolar gas packing fraction (φ) in lungs.** The φ values were summarized in **Table 1** in the manuscript.

**Supplementary Table S1.**

**Sphericity of 120 individual alveoli (μm) in 3 live mice lungs and 3 fixed mice lungs.**

Here S is the surface area of the alveoli, V is the volume of the alveoli, and φ is the sphericity of individual alveoli (φ of the sphere = 1). φ = 0.90 ± 0.02 (mean ± s.d.) for the live lungs and φ = 0.79 ± 0.04 (mean ± s.d.) for the fixed lungs were taken from X-ray microtomography (*P*-value = 2.8 × 10^-67^). Every single measurement is not from the same alveolus.

| **Alveoli in the live lung** | | | **Alveoli in the fixed lung** | | |
| --- | --- | --- | --- | --- | --- |
| **S (μm^2^)** | **V (μm^3^)** | $\boldsymbol{\varphi}$ | **S (μm^2^)** | **V (μm^3^)** | $\boldsymbol{\varphi}$ |
| 18,538 | 217,711 | 0.94 | 7,104 | 41,131 | 0.81 |
| 27,600 | 358,687 | 0.88 | 7,534 | 45,745 | 0.82 |
| 25,477 | 334,170 | 0.91 | 4,087 | 18,538 | 0.83 |
| 13,486 | 127,601 | 0.91 | 4,445 | 19,659 | 0.79 |
| 19,485 | 213,130 | 0.89 | 6,179 | 32,664 | 0.80 |
| 21,032 | 240,148 | 0.89 | 6,806 | 38,148 | 0.81 |
| 22,410 | 265,826 | 0.89 | 5,666 | 29,350 | 0.81 |
| 39,887 | 637,394 | 0.90 | 4,824 | 20,565 | 0.75 |
| 23,939 | 300,497 | 0.91 | 4,237 | 19,217 | 0.82 |
| 11,460 | 97,056 | 0.89 | 4,655 | 20,718 | 0.78 |
| 26,027 | 333,800 | 0.89 | 4,527 | 21,334 | 0.82 |
| 18,301 | 197,968 | 0.90 | 3,413 | 14,173 | 0.83 |
| 30,021 | 425,591 | 0.91 | 3,674 | 15,963 | 0.83 |
| 18,024 | 195,911 | 0.91 | 4,019 | 13,667 | 0.69 |
| 17,608 | 190,945 | 0.91 | 3,843 | 16,812 | 0.83 |
| 42,554 | 714,185 | 0.91 | 3,966 | 16,787 | 0.80 |
| 25,939 | 324,395 | 0.88 | 3,486 | 13,433 | 0.78 |
| 32,307 | 499,784 | 0.94 | 2,640 | 9,194 | 0.80 |
| 33,283 | 462,991 | 0.87 | 4,127 | 18,258 | 0.81 |
| 22,900 | 267,553 | 0.88 | 4,476 | 21,879 | 0.85 |
| 35,681 | 546,110 | 0.91 | 5,175 | 26,187 | 0.82 |
| 15,184 | 145,488 | 0.88 | 3,973 | 16,565 | 0.79 |
| 14,847 | 153,631 | 0.93 | 6,229 | 35,370 | 0.84 |
| 17,272 | 192,396 | 0.93 | 4,079 | 15,778 | 0.75 |
| 21,279 | 267,826 | 0.94 | 5,598 | 26,468 | 0.77 |
| 17,098 | 180,474 | 0.90 | 6,380 | 33,608 | 0.79 |
| 52,251 | 975,742 | 0.91 | 2,745 | 10,289 | 0.83 |
| 9,708 | 77,658 | 0.91 | 13,954 | 117,746 | 0.83 |
| 37,323 | 593,185 | 0.91 | 9,820 | 57,038 | 0.73 |
| 34,856 | 517,865 | 0.89 | 5,101 | 25,071 | 0.81 |
| 17,499 | 178,859 | 0.88 | 4,384 | 15,968 | 0.70 |
| 26,501 | 348,548 | 0.90 | 5,324 | 25,931 | 0.80 |
| 18,426 | 211,979 | 0.93 | 8,102 | 49,003 | 0.80 |
| 12,856 | 118,131 | 0.91 | 5,142 | 24,909 | 0.80 |
| 40,190 | 652,940 | 0.91 | 7,173 | 41,030 | 0.80 |
| 16,812 | 176,980 | 0.91 | 2,799 | 10,044 | 0.80 |
| 9,409 | 70,666 | 0.88 | 2,405 | 7,340 | 0.76 |
| 9,220 | 74,970 | 0.93 | 5,441 | 26,649 | 0.79 |
| 25,547 | 334,008 | 0.91 | 2,912 | 11,398 | 0.84 |
| 19,344 | 220,481 | 0.91 | 6,914 | 37,541 | 0.78 |
| 37,399 | 547,001 | 0.86 | 3,678 | 15,882 | 0.83 |
| 14,761 | 152,684 | 0.94 | 5,416 | 25,984 | 0.78 |
| 14,409 | 145,006 | 0.93 | 4,197 | 17,200 | 0.77 |
| 23,022 | 292,985 | 0.93 | 3,580 | 15,316 | 0.83 |
| 24,820 | 311,439 | 0.90 | 2,914 | 9,703 | 0.76 |
| 32,733 | 467,043 | 0.89 | 4,901 | 21,148 | 0.75 |
| 47,711 | 828,518 | 0.89 | 4,019 | 15,490 | 0.75 |
| 21,764 | 263,601 | 0.91 | 2,539 | 7,348 | 0.72 |
| 10,919 | 95,730 | 0.93 | 1,804 | 4,773 | 0.76 |
| 16,308 | 177,240 | 0.94 | 3,290 | 11,027 | 0.73 |
| 14,363 | 141,169 | 0.91 | 1,179 | 2,758 | 0.81 |
| 20,643 | 247,101 | 0.92 | 1,979 | 5,898 | 0.80 |
| 10,795 | 95,344 | 0.93 | 1,921 | 5,775 | 0.81 |
| 17,273 | 186,369 | 0.91 | 4,001 | 16,369 | 0.78 |
| 17,561 | 193,619 | 0.92 | 3,561 | 13,961 | 0.79 |
| 15,528 | 146,272 | 0.86 | 969 | 1,811 | 0.74 |
| 14,245 | 132,686 | 0.88 | 1,849 | 5,062 | 0.77 |
| 10,812 | 91,662 | 0.91 | 2,722 | 10,429 | 0.85 |
| 13,386 | 121,721 | 0.89 | 3,425 | 12,460 | 0.76 |
| 10,434 | 88,820 | 0.92 | 2,836 | 9,014 | 0.74 |
| 10,920 | 93,662 | 0.91 | 3,619 | 14,017 | 0.78 |
| 11,591 | 99,150 | 0.89 | 2,978 | 11,251 | 0.82 |
| 25,959 | 338,851 | 0.91 | 1,902 | 5,659 | 0.81 |
| 18,617 | 206,533 | 0.91 | 2,133 | 6,880 | 0.82 |
| 13,655 | 126,601 | 0.89 | 1,986 | 6,224 | 0.82 |
| 8,682 | 61,148 | 0.86 | 3,412 | 14,294 | 0.83 |
| 9,519 | 72,482 | 0.88 | 3,271 | 11,696 | 0.76 |
| 18,481 | 206,653 | 0.91 | 4,479 | 15,485 | 0.67 |
| 17,318 | 185,514 | 0.91 | 2,769 | 10,083 | 0.82 |
| 9,819 | 80,665 | 0.92 | 3,485 | 15,299 | 0.86 |
| 24,762 | 314,589 | 0.90 | 3,597 | 15,332 | 0.83 |
| 14,796 | 140,888 | 0.88 | 6,036 | 33,361 | 0.83 |
| 15,996 | 150,194 | 0.85 | 2,890 | 11,334 | 0.84 |
| 44,462 | 701,158 | 0.86 | 5,579 | 24,624 | 0.73 |
| 36,505 | 552,572 | 0.89 | 2,545 | 8,682 | 0.80 |
| 26,512 | 322,844 | 0.86 | 3,129 | 11,691 | 0.80 |
| 40,378 | 644,606 | 0.89 | 2,669 | 8,208 | 0.74 |
| 23,860 | 302,232 | 0.91 | 4,187 | 16,841 | 0.76 |
| 34,350 | 514,940 | 0.90 | 1,700 | 4,688 | 0.80 |
| 22,681 | 272,120 | 0.90 | 4,538 | 22,628 | 0.85 |
| 41,975 | 693,561 | 0.90 | 5,388 | 29,191 | 0.85 |
| 30,088 | 418,357 | 0.90 | 7,163 | 37,759 | 0.76 |
| 38,935 | 635,598 | 0.92 | 3,123 | 12,014 | 0.81 |
| 20,168 | 236,471 | 0.92 | 4,553 | 22,912 | 0.86 |
| 67,667 | 1,432,143 | 0.91 | 3,029 | 11,432 | 0.81 |
| 22,799 | 282,804 | 0.91 | 4,076 | 15,359 | 0.73 |
| 28,447 | 402,085 | 0.93 | 4,614 | 20,886 | 0.79 |
| 29,725 | 399,312 | 0.88 | 4,131 | 15,120 | 0.72 |
| 21,192 | 252,996 | 0.91 | 4,126 | 18,285 | 0.81 |
| 40,698 | 647,375 | 0.89 | 2,961 | 11,484 | 0.83 |
| 16,385 | 174,270 | 0.92 | 5,985 | 31,823 | 0.81 |
| 21,105 | 243,406 | 0.89 | 4,838 | 22,432 | 0.80 |
| 58,002 | 1,112,985 | 0.90 | 3,801 | 16,684 | 0.83 |
| 17,019 | 173,547 | 0.88 | 2,969 | 11,095 | 0.81 |
| 20,514 | 232,831 | 0.89 | 2,641 | 8,978 | 0.79 |
| 33,532 | 498,030 | 0.91 | 6,932 | 35,600 | 0.75 |
| 22,312 | 255,863 | 0.87 | 5,172 | 23,481 | 0.77 |
| 62,984 | 1,239,845 | 0.89 | 4,028 | 17,021 | 0.79 |
| 25,227 | 320,676 | 0.90 | 9,775 | 68,332 | 0.83 |
| 39,093 | 606,568 | 0.89 | 5,293 | 26,459 | 0.81 |
| 35,839 | 551,961 | 0.91 | 3,417 | 13,739 | 0.81 |
| 15,045 | 146,418 | 0.89 | 5,372 | 27,026 | 0.81 |
| 19,998 | 227,554 | 0.90 | 5,207 | 25,512 | 0.80 |
| 22,251 | 268,750 | 0.91 | 4,908 | 23,026 | 0.80 |
| 23,331 | 293,709 | 0.92 | 2,173 | 7,140 | 0.83 |
| 13,713 | 136,708 | 0.94 | 3,137 | 10,804 | 0.75 |
| 20,193 | 234,627 | 0.91 | 3,406 | 12,557 | 0.77 |
| 35,383 | 533,682 | 0.90 | 3,838 | 15,381 | 0.78 |
| 32,721 | 474,369 | 0.90 | 9,177 | 59,840 | 0.81 |
| 21,985 | 256,838 | 0.89 | 5,077 | 21,843 | 0.74 |
| 42,285 | 699,216 | 0.90 | 2,229 | 7,441 | 0.83 |
| 48,916 | 859,868 | 0.89 | 5,314 | 27,107 | 0.82 |
| 44,873 | 793,890 | 0.92 | 3,732 | 14,462 | 0.77 |
| 39,952 | 615,170 | 0.88 | 4,943 | 24,441 | 0.82 |
| 14,324 | 138,034 | 0.90 | 2,634 | 8,582 | 0.77 |
| 23,092 | 289,850 | 0.92 | 3,729 | 15,741 | 0.81 |
| 16,543 | 178,856 | 0.93 | 4,949 | 24,957 | 0.83 |
| 27,272 | 351,888 | 0.88 | 2,340 | 8,028 | 0.83 |
| 29,522 | 398,066 | 0.89 | 4,895 | 20,019 | 0.73 |
| 23,296 | 288,598 | 0.91 | 4,480 | 17,958 | 0.74 |

**Supplementary Table S2.**

**Diameters of 120 individual alveoli (**$\boldsymbol{\mu m}$**) in 3 live mice lungs and 3 fixed mice lungs.**

*D*: diameter of alveoli. *D* = 81.6 ± 18.2 $\mu m$ (mean ± s.d.) for a live lungs and *D* = 35.9 ± 7.2 $\mu m$ (mean ± s.d.) for a fixed lungs (*P*-value$=$ 3.0 × 10^-57^). Every single measurement is not from the same alveolus.

| ***D (***$\boldsymbol{\mu m)}$ | |
| --- | --- |
| ***Live*** | ***Fixed*** |
| 73.7 | 45.2 |
| 90.2 | 46.8 |
| 88.6 | 33.5 |
| 70.3 | 33.7 |
| 70.1 | 41.0 |
| 81.2 | 43.2 |
| 80.5 | 35.7 |
| 112.3 | 40.1 |
| 95.2 | 39.5 |
| 57.9 | 35.2 |
| 87.7 | 30.4 |
| 75.2 | 32.5 |
| 95.8 | 32.1 |
| 71.5 | 30.1 |
| 70.4 | 33.3 |
| 108.5 | 33.5 |
| 89.2 | 31.0 |
| 98.7 | 28.9 |
| 91.2 | 33.9 |
| 78.9 | 36.1 |
| 95.2 | 38.5 |
| 68.0 | 36.2 |
| 68.2 | 45.7 |
| 72.3 | 30.3 |
| 83.2 | 40.5 |
| 69.9 | 45.8 |
| 130.1 | 36.1 |
| 55.3 | 68.9 |
| 99.7 | 50.4 |
| 102.4 | 37.0 |
| 68.5 | 41.1 |
| 79.5 | 45.2 |
| 71.1 | 54.8 |
| 58.2 | 41.8 |
| 105.2 | 47.6 |
| 68.6 | 30.2 |
| 52.8 | 31.7 |
| 48.7 | 42.6 |
| 90.1 | 33.8 |
| 86.7 | 49.7 |
| 101.5 | 31.2 |
| 66.3 | 36.7 |
| 65.2 | 32.0 |
| 82.4 | 30.8 |
| 84.1 | 26.5 |
| 96.3 | 34.3 |
| 116.5 | 30.9 |
| 79.6 | 24.1 |
| 56.8 | 20.9 |
| 69.7 | 27.6 |
| 70.6 | 28.4 |
| 83.9 | 27.4 |
| 62.7 | 29.3 |
| 76.9 | 35.5 |
| 77.8 | 33.9 |
| 71.4 | 29.1 |
| 69.3 | 28.3 |
| 61.9 | 31.1 |
| 67.5 | 32.8 |
| 61.4 | 29.8 |
| 62.3 | 33.9 |
| 63.4 | 31.8 |
| 92.5 | 30.1 |
| 79.3 | 29.6 |
| 68.3 | 27.8 |
| 54.9 | 34.1 |
| 57.7 | 32.2 |
| 79.4 | 34.9 |
| 76.8 | 30.8 |
| 59.6 | 34.8 |
| 90.4 | 34.8 |
| 70.6 | 43.9 |
| 72.0 | 31.9 |
| 116.2 | 40.1 |
| 107.8 | 29.5 |
| 91.1 | 32.2 |
| 113.2 | 29.0 |
| 89.3 | 35.8 |
| 105.4 | 24.8 |
| 86.4 | 39.1 |
| 109.8 | 38.2 |
| 92.8 | 41.6 |
| 106.7 | 28.4 |
| 76.7 | 35.2 |
| 139.9 | 27.9 |
| 81.4 | 30.8 |
| 91.6 | 34.2 |
| 91.4 | 30.7 |
| 78.5 | 32.7 |
| 107.3 | 28.0 |
| 60.3 | 44.3 |
| 68.5 | 40.0 |
| 119.6 | 36.7 |
| 60.2 | 32.7 |
| 67.3 | 30.8 |
| 89.3 | 45.8 |
| 69.8 | 40.5 |
| 124.3 | 36.9 |
| 75.9 | 55.7 |
| 96.0 | 42.0 |
| 92.8 | 34.7 |
| 56.4 | 42.2 |
| 66.7 | 41.5 |
| 71.1 | 40.3 |
| 73.5 | 28.9 |
| 54.9 | 32.4 |
| 67.5 | 33.8 |
| 91.6 | 35.9 |
| 87.8 | 53.5 |
| 69.9 | 39.7 |
| 101.1 | 29.2 |
| 109.0 | 42.3 |
| 105.9 | 35.2 |
| 96.5 | 41.0 |
| 55.1 | 30.4 |
| 73.1 | 36.1 |
| 60.9 | 41.3 |
| 78.6 | 29.8 |
| 82.3 | 38.7 |
| 73.0 | 37.5 |
